# Defining diagnostic cutoffs in neurological patients for serum very long chain fatty acids (VLCFA) in genetically confirmed X-Adrenoleukodystrophy

**DOI:** 10.1038/s41598-020-71248-8

**Published:** 2020-09-15

**Authors:** Tim W. Rattay, Maren Rautenberg, Anne S. Söhn, Holger Hengel, Andreas Traschütz, Benjamin Röben, Stefanie N. Hayer, Rebecca Schüle, Sarah Wiethoff, Lena Zeltner, Tobias B. Haack, Alexander Cegan, Ludger Schöls, Erwin Schleicher, Andreas Peter

**Affiliations:** 1 Department of Neurology and Hertie-Institute for Clinical Brain Research, University of Tübingen, Tübingen, Germany; 2 German Center of Neurodegenerative Diseases (DZNE), Tübingen, Germany; 3 Institute of Medical Genetics and Applied Genomics, University of Tübingen, Tübingen, Germany; 4 Center of Rare Diseases (ZSE), University of Tübingen, Tübingen, Germany; 5 Department of Biological and Biochemical Sciences, Faculty of Chemical Technology, University of Pardubice, Pardubice, Czech Republic; 6 Institute for Clinical Chemistry and Pathobiochemistry/Central Laboratory, University of Tübingen, Hoppe-Seyler-Str. 3, 72076 Tübingen, Germany; 7 German Center for Diabetes Research (DZD), Tübingen, Germany; 8 Institute for Diabetes Research and Metabolic Diseases (IDM) of the Helmholtz Centre Munich at the University of Tübingen, Tübingen, Germany

**Keywords:** Genetics of the nervous system, Fatty acids, Analytical biochemistry, Neurological disorders, Diagnostic markers, Neurology

## Abstract

X-linked Adrenoleukodystrophy (X-ALD) is caused by mutations in the *ABCD1* gene resulting in the accumulation of very long chain fatty acids (VLCFA). X-ALD is the most common peroxisomal disorder with adult patients (male and female) presenting with progressive spastic paraparesis with bladder disturbance, sensory ataxia with impaired vibration sense, and leg pain. 80% of male X-ALD patients have an adrenal failure, while adrenal dysfunction is rare in women with X-ALD. The objective of this study was to define optimal serum VLCFA cutoff values in patients with X-ALD-like phenotypes for the differentiation of genetically confirmed X-ALD and Non-X-ALD individuals. Three groups were included into this study: a) X-ALD cases with confirmed *ABCD1* mutations (n = 34) and two Non-X-ALD cohorts: b) Patients with abnormal serum VCLFA levels despite negative testing for *ABCD1* mutations (n = 15) resulting from a total of 1,953 VLCFA tests c) Phenotypically matching patients as Non-X-ALD controls (n = 104). Receiver operating curve analysis was used to optimize VLCFA cutoff values, which differentiate patients with genetically confirmed X-ALD and Non-X-ALD individuals. The serum concentration of C26:0 was superior to C24:0 for the detection of X-ALD. The best differentiation of Non-X-ALD and X-ALD individuals was obtained with a cutoff value of < 1.0 for the C24:0/C22:0 ratio resulting in a sensitivity of 97%, a specificity of 94.1% and a positive predictive value (PPV) of 83.8% for true X-ALD. Our findings further suggested a cutoff of < 0.02 for the ratio C26:0/C22:0 leading to a sensitivity of 90.9%, a specificity of 95.0%, and a PPV of 80.6%. Pearson correlation indicated a significant positive association between total blood cholesterol and VLCFA values. Usage of serum VLCFA are economical and established biomarkers suitable for the guidance of genetic testing matching the X-ALD phenotype. We suggest using our new optimized cutoff values, especially the two ratios (C24:0/C22:0 and C26:0/C22:0), in combination with standard lipid profiles.

## Background

X-linked Adrenoleukodystrophy (X-ALD) is the most common peroxisomal disorder worldwide^1,2^ and caused by mutations in the X-chromosomal *ABCD1* gene^3^. Impaired peroxisomal beta-oxidation leads to the accumulation of very long chain fatty acids (VLCFA > 22 carbon atoms) in blood and a variety of tissues (e.g. brain white matter, spinal cord, and adrenal cortex). Adults usually develop an adrenomyeloneuropathy (AMN) phenotype characterized by progressive spastic paraparesis with bladder disturbance, sensory ataxia with impaired vibration sense, leg pain, and adrenal failure in male patients. about one-third of cases, young boys present with signs and symptoms that are due to the onset of cerebral ALD. Nerve conduction studies often reveal multifocal demyelination^4^ with detectable peripheral nerve abnormalities by high-resolution ultrasound^5^. The AMN phenotype is an important differential diagnosis to hereditary spastic paraplegias (HSP)^6,7^, which are clinically and genetically very heterogeneous (80+ genes are described—see^8^). The accumulation of VLCFA in the blood can be used diagnostically in X-ALD from either plasma or serum. Elevated levels of cerotic acid (C26:0) [overall reference range 0.15–1.9 µmol/l] and the ratios C24:0/C22:0 [0.32–1.19] and C26:0/C22:0 [0.0–0.03] are used for diagnostic purposes (see cutoff level comparison in Table 1 and as previously discussed by Moser et al.^9^). Moser et al.^10^ introduced a discriminant function based on the measurement of C26:0 and the two ratios C26/22 and C24/22 to enhance the distinction between values obtained for X-ALD males and control males and X-ALD females and control females. Our diagnostic laboratory does not currently use this function. An increasingly important alternative for the determination of VLCFA is the use of 1-hexacosanosyl-2-lyso-sn-3-glycero-phosphatidylcholine (26:0-lyso-PC) in dried blood spots (Hubbard WC et al., 2009), being sensitive for females (Huffnagel et al. 2017) as well. Preanalytical issue need to be considered e.g. VLCFA values are partially influenced by current diet e.g. by ketogenic (shown by Moser et al.^10^ and others) or vegetarian diet, with vegetarians potentially showing elevated values (Table 1). These reports and our observation that elevated serum lipids may influence VLCFA values led us to simultaneously determine the lipid profile in samples with requests for VLCFA analysis to evaluate this factor and improve the diagnostic workup.

**Table 1:** Reference values of very long chain fatty acids in selected publications.

Reference	Moser 1999^10^		Ronghe 2002^26^	Horn 2013^27^	Martinez 1994^16^*		Streck 2000^28^	Stellaard 1990^29^	Morell 2010^13^	Lagerstedt 2001^30^	
Patients	n = 1084 (hemi., ♂)	n = 379 (het., ♀)	n.r.	n.r.	n = 26		n.a.	n.a.	n.a.	n = 1	Overall ranges
C26:0 [µmol/l]	2.98 ± 0.34	1.77 ± 1.01	n.r.	n.r.	4.9 ± 2.4		n.a.	n.a.	n.a.	2.6	[0.76–7.30]
ratio C24:0/C22:0	1.49 ± 0.45	1.09 ± 0.34	n.r.	n.r.	1.48 ± 0.31		n.a.	n.a.	n.a.	1.46	[0.75–1.94]
ratio C26:0/C22:0	0.07 ± 0.04	0.03 ± 0.02	n.r.	n.r.	0.07 ± 0.03		n.a.	n.a.	n.a.	0.04	[0.01–0.11]
*ABCD1* mutations	Not tested		Not tested	Partially tested	Not tested		n.a.	n.a.	n.a.	Not tested	
Controls	n = 11,048 (♂)	n = 7331 (♀)	n.r.	n.r.	n = 27 (omni.)	n = 4 (veg.)	n = 22	n = 21	n.r. (“in house”)	n = 43	Overall reference ranges
C26:0 [µmol/l]	0.66 ± 0.38	0.66 ± 0.38	0.33–1.39	0.3–1.0	1.3 ± 0.4	2.2 ± 0.9	0.15–0.39	0.22–1.31	0.3–1.9	0.3–1.3	[0.15–1.9] with veg.[0.28–3.1]^16^
C24:0/C22:0	0.86 ± 0.22	0.85 ± 0.20	0.32–0.90	0.49–0.91	0.82 ± 0.08	1.08 ± 0.11	0.66–0.94	0.32–1.19	0.3–1.1	n.r.	[0.32–1.19] with veg.[0.28–1.19]^16^
C26:0/C22:0	0.01 ± 0.02	0.01 ± 0.01	0.0–0.03	0.006–0.021	0.02 ± 0.01	0.02 ± 0.01	0.005–0.012	0.003–0.021	0.02–0.025	n.r.	[0–0.03]

This table presents VLCFA values previously published for adult X-ALD patients (upper half) and healthy controls (lower half), the columns represent different publications. For the X-ALD patients, there is also listed if ABCD1 mutations were tested in these studies or not (see the center row of the table). *Martinez et al. did not differentiate between X-ALD and other peroxisomal diseases within the mentioned ranges. Data are presented as mean ± SD (standard deviation). C26:0 concentration shown is µmol/l (data was converted to match the unified unity µmol/l using a molecular weight of 396.702 for C26:0). The reference range was calculated using the mean ± SD and then taking from all reference values the lowest and the highest value each.

hemi., hemizygous; het., heterozygous; n.a., not applicable; n.r., not reported; veg., vegetarian; omni., omnivores.

VLCFA levels have previously been measured in large groups of X-ALD patients with the clinical diagnosis of X-ALD and healthy controls (for selected references see Table 1). However, reference ranges vary considerably between the reports: e.g. for C26:0: 0.66 ± 038^10^; 1.3 ± 0.4^16^ and 0.3–1.9^13^ µmol/L and for the ratio C26:0/C22:0: 0.01 ± 0.02^10^; 0.02 ± 0.01^16^ and 0.02–0.025^13^, respectively, just to mention the most differing reference ranges. The VLCFA values from patients are interpreted using reference ranges obtained from neurological healthy controls. However, reference ranges (mean ± 2SD), which cover 95% of the values of the reference cohort) are not optimal to assess results obtained from rare diseases because, by definition, 2.5% of the healthy controls have values above two standard deviations. This means that for any 1000 healthy controls tested, 25 have elevated VLCFA values. This number of false-positives is too large to allow screening for X-ALD in neurologically affected patients with an incidence at the birth of ~ 1:17,000^2^. Instead of using reference ranges, the establishment of a threshold or cutoff is more helpful for the clinician in the case of X-ALD: 2.5% out of 17,000 healthy individuals = 425 will show elevated VLCFA, i.e. far too many false-positive values will be obtained.

Up to date assessment of VLCFA cutoffs in patients with a mimicking phenotype as well as in genetically confirmed X-ALD cases is missing. We set out to a) use receiver operating curve (ROC) analysis to optimize VLCFA cutoff values to differentiate patients with genetically confirmed X-ALD and Non-X-ALD individuals with X-ALD-like phenotypes or other causes of VLCFA abnormalities and b) investigate a possible effect of serum lipid levels on VLCFA.

## Materials and methods

### Study rationale

Reference ranges for very long chain fatty acids vary in the literature (compare Table 1). The current reference ranges have limited value for screening due to overlap in the ranges for healthy controls and X-ALD patients and due to low specificity: False-positive findings occur in any VLCFA (C24:0, C:26:0 and the ratios C24:0/C22:0 and C26:0/C22:0).

We set out to optimize cutoff values for VLCFA and to estimate the sensitivity and specificity of each parameter. Therefore, we used a cohort of genetically confirmed X-ALD patients (not to miss heterozygote females with normal VLCFA levels), phenotypically matched Non-X-ALD cases, and cases with elevated VLCFA levels (false-positive; *ABCD1* mutation-negative).

### Patient cohort

**Figure 1:**
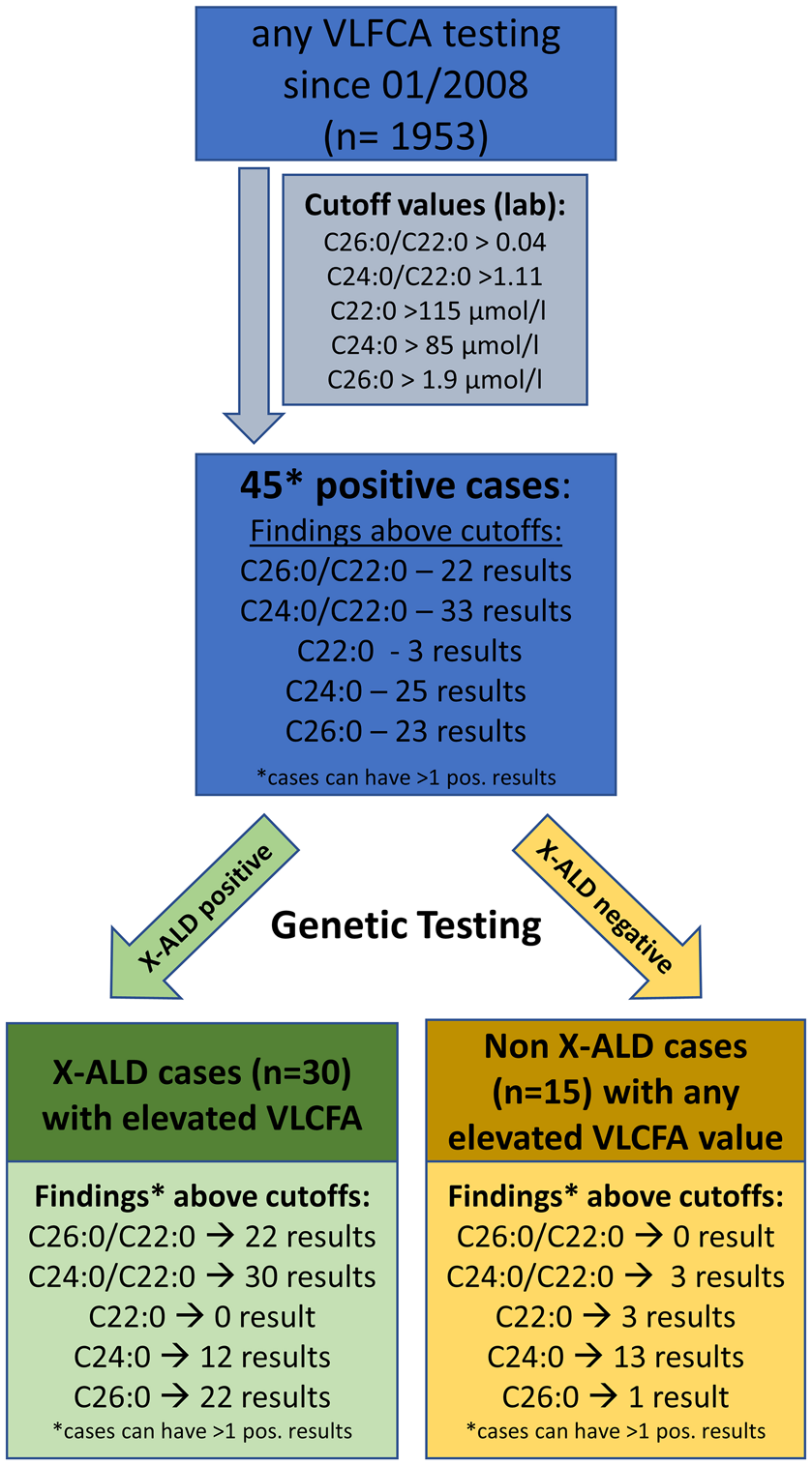
Flowchart of VLCFA testing. Since 01/2008, a total number of n = 1953 VLCFA tests were performed in the central laboratory of the University Hospital Tübingen in patients where X-ALD was considered an appropriate differential diagnosis. Thereof 45 patients had at least > 1 positive VLCFA results according to the cutoffs mentioned in the light blue box. By genetic testing, the 45 positive cases were grouped into two groups, the X-ALD positive cases (n = 30) and the X-ALD negative cases (n = 15). Cases with confirmed *ABCD1*-mutations (compare supplementary Table 1) were considered as X-ALD cases, and the Non-X-ALD cases consisted of cases without *ABCD1*-mutation or another genetically proven disorder (compare Table 2). Since, in many cases > 1 VLCFA result was positive, the specified findings in either the lighter green or light yellow box do not sum up to the number of cases specified above.

Non-hemolytic sera from patients with typical symptoms of X-ALD were tested in the Institute for Clinical Chemistry and Pathobiochemistry at the University Hospital Tübingen using the current VLCFA cutoff values (see Fig. 1 for details). From 01/2008 till 01/2020, 1953 patients (nine minors) were analyzed and included in the study. For eleven participants, consecutive VLCFA measurements (up to eight visits per patient) were available. Those were analyzed separately; see supplementary material for results. Genetic testing for the majority of patients was performed in the Department of Medical Genetics, University of Tübingen, either as single-gene analysis by PCR or by next-generation sequencing techniques, e.g. panel-, exome- or genome-based approaches. Some patients had prior external genetic confirmation of their diagnosis.

### Ethics approval and consent to participate

This study was carried out in compliance with the Helsinki Declaration and approved by the Institutional Review Board of the University of Tübingen, reference number (690/2011BO1). Nine minors were included in this study. Written informed consent was obtained from all patients or caregivers/legal guardians if applicable.

### Analysis of very long chain fatty acids (VLCFA)

Spontaneous (fasted and unfasted) blood samples were immediately centrifuged, and serum aliquots were stored at 4 °C and analyzed within two days, or the serum sample was frozen and stored at − 80 °C. The determination of VLCFA was performed according to Lepage and Roy with modifications^11^. For analysis, 0.1 ml aliquots from patients' serum samples were placed in Pyrex tubes and 2 ml methanol/toluene mixture (4:1, vol/vol) containing 10 mg/l cis-13,16,19-docosatrienoic acid as an internal standard were added. A small stirring bar was added and trans-esterification was achieved by slow addition (~ 1 min) of 0.2 ml acetyl chloride to the samples and heating for 1 h at 100 °C. The cold sample was neutralized with 6% K2CO3, cleared by centrifugation, and the upper phase was concentrated to 180 μl under a stream of nitrogen. Only analytical grade reagents were used. The fatty acid methyl esters were transferred to screw-capped vials and analyzed by gas chromatography. Chromatographic separation was performed by gas chromatography (Agilent 7890A, Böblingen, Germany) equipped with a 50 m HT 80 column, internal diameter 0.22 mm from SGE (Langwehe, Germany) and quantified by flame ionization detector. The injection port temperature was 230 °C and 250 °C for the detector. For analysis, 1 µl of the sample was injected (split ratio 1:10), the column temperature was 130 °C, and held constant for 2 min. Then the temperature was raised by 2 °C/min till 160 °C, kept constant for 1 min, then raised by 1 °C/min till 260 °C, and then the temperature was held constant for 10 min. The retention time was 93, 108, and 123 min for C22:0, C24:0, and C26:0, respectively.

The calibration was performed with the help of a calibration curve with commercially available fatty acids (Merck, Darmstadt, Germany), which were treated the same way as patient samples. We used cis-13,16,19-docosatrienoic acid as internal standard since this fatty acid was not found in human samples while previously recommended fatty acids with odd carbon numbers e,g, C 15:0, and C17:0, and others do occur in human serum samples. For quality control, pooled serum of 20 healthy subjects with concentrations of C22:0, C24:0, and C26:0 of 65, 56, and 0.3 µmol/l was prepared and spiked with C26:0 to yield a concentration of 1.3 µmol/l (QC 1). To obtain a QC with elevated values, the pooled serum was spiked with all fatty acids of interest to yield 228, 130, and 2.8 µmol/l for C C22:0, C24:0, and C26:0, respectively (QC 2). Both quality controls were analyzed every run together with patients' samples. The coefficient of variations was 2.25, 2.8 and 9.4% for C22:0, C24:0, C26:0 (QC 1) and 4.96, 3.87 and 5.29% for C22:0, C24:0, C26:0 for control 2. Both patient samples and controls were cross-checked with a reference laboratory at least yearly to verify the accuracy of the analysis. All patient samples with concentrations of C26:0 > 0.5 µmol/l were analyzed a second time independently, not to miss an elevated VLCFA. In any case, we found that the repeated analysis yielded results differing by less than 8% from the first analysis.

### Routine lipid analysis

For n = 90 cases, routine lipid analysis including total cholesterol, high-density lipoprotein (HDL), low-density lipoprotein (LDL), and triglyceride levels was analyzed to evaluate a possible association of VLCFA levels and serum lipid levels. The ADVIA XPT clinical chemistry analyzer (Siemens Healthineers, Eschborn, Germany) was used for routine lipid analysis. The coefficient of variation from day to day was below 5% for all analytes.

### Defining new diagnostic cutoffs

**Figure 2:**
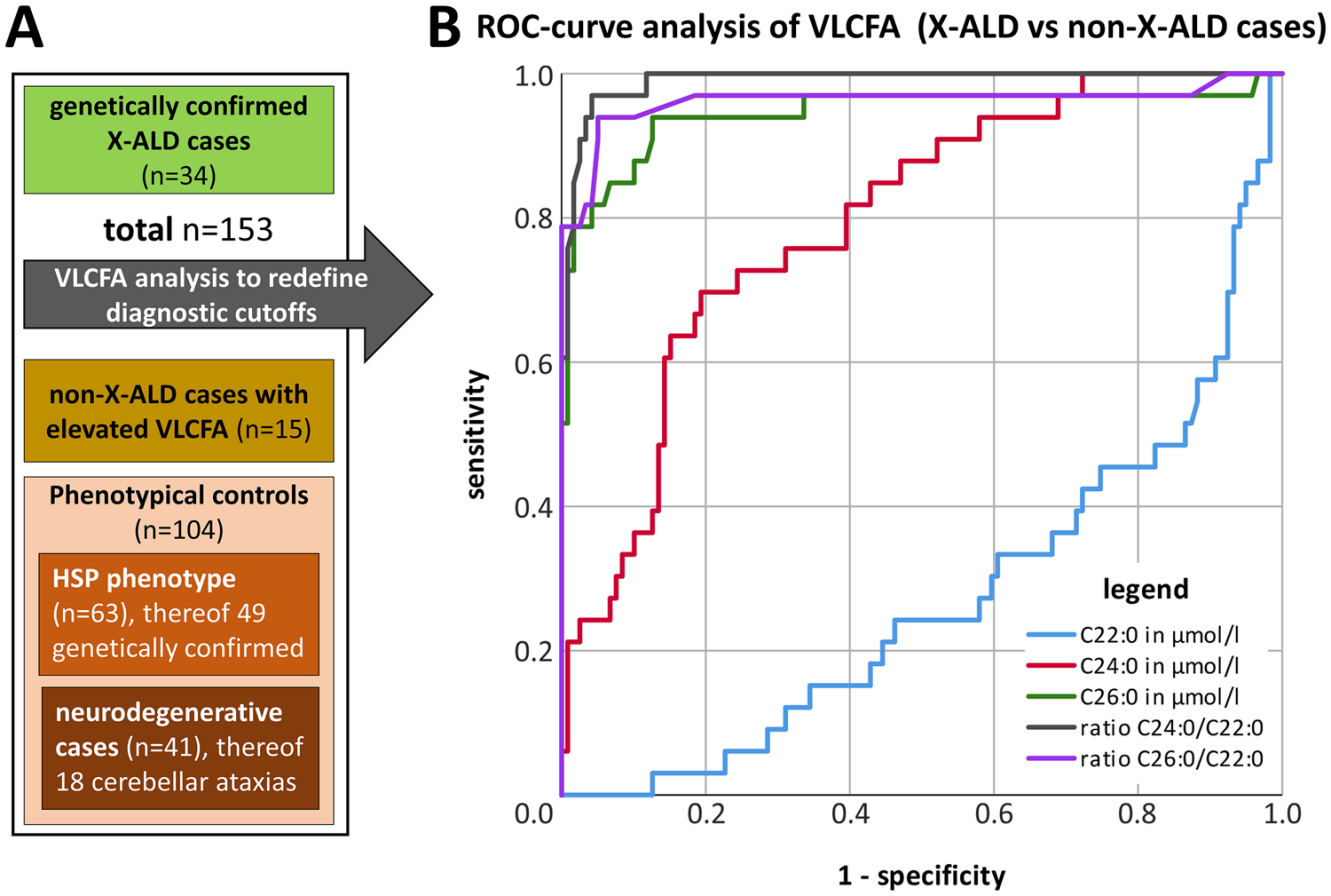
VLCFA evaluation in phenotypically similar patients. (**A**) Using the same dataset, the VLCFA values of phenotypically similar patients (black box—n = 153) were evaluated to redefine diagnostic cutoffs. The positive VLCFA cases (n = 45; 30 X-ALD and 15 Non-X-ALD) identified via the screening since 01/2008 (compare Fig. 1) were combined with four X-ALD patients with VLCFA values below the previously used cutoffs totaling 34 X-ALD cases. Phenotypical controls (n = 104) matching the Non-X-ALD cases phenotypically with elevated VLCFA (compare Table 3) were chosen from the same dataset. (**B**) ROC- analysis of X-ALD versus Non-X-ALD cases was performed using all five VLCFA measures (color coding see legend within the Figure) for the 153 phenotypically similar cases (as specified in the “Methods” section—see redefining diagnostic cutoffs). Sensitivity and specificity were highest for the C24:0/C22:0 ratio followed by the C26:0/C22:0 ratio and the C26:0 absolute value.

To evaluate and define new diagnostic cutoffs, VLCFA values of phenotypically similar patients (n = 153) were evaluated (see Fig. 2) from the dataset. In addition to the 45 positive VLCFA cases (30 X-ALD and 15 Non-X-ALD (see Table 2 and Fig. 1), four further X-ALD patients were added to the cohort with VLCFA values below the previously used cutoffs. Resulting in 34 genetically confirmed X-ALD cases (four minors; all mutations are specified in Supplementary Table 1). The phenotypical control group (n = 104, compare Fig. 2A) consisted of: a) Patients with the clinical diagnosis of hereditary spastic paraplegia (n = 63). Thereof, 73% (n = 49) had a genetically confirmed HSP diagnosis (one minor) and 14 patients remained genetically unsolved despite exome analysis. b) Other matched neurodegenerative cases (n = 41), including cerebellar ataxia cases (n = 18) and other complex neurodegenerative cases (four minors) including leukoencephalopathies (n = 23).

**Table 2:** Synopsis of patients with elevated VLCFA without X-ALD/*ABCD1* gene mutations.

ID	diagnosis	♀/♂	age [years]	behenic acid (C22:0) [µmol/l]	lignoceric acid (C24:0) [µmol/l]	cerotic acid (C26:0) [µmol/l]	ratio C24:0/C22:0	ratio C26:0/C22:0	total cholesterol [mg/dl]	TG [mg/dl]	HDL [mg/dl]	LDL [mg/dl]	Comments
65	SPG4	**♀**	58	104.7	**88.3**	0.33	0.84	0.003	**247**	**154**	65	160	
69	SPG4	**♂**	40	105.5	**99.4**	0.44	0.94	0.004	**283**	**502**	55	171	
76	SPG39	**♂**	54	105.2	**103.2**	0.43	0.98	0.004	**298**	118	74	215	
77	SPG31	**♂**	60	33.4	48.2	0.39	**1.29**	0.012	n.a.	n.a.	n.a.	n.a.	Tested neg. for *ABCD1* mut
112	WMD	**♀**	79	**140.6**	**96.3**	0.96	0.69	0.01	n.a.	n.a.	n.a.	n.a.	
113	HSP	**♂**	59	93.0	**101.8**	1.16	1.09	0.012	**275**	**289**	67	175	(Normal VLCFA in follow-ups) tested neg. for *ABCD1* mut
115	HSP	**♀**	72	**115.9**	**92.4**	0.61	0.80	0.01	n.a.	n.a.	n.a.	n.a.	Tested neg. for *ABCD1* mut
119	HSP	**♀**	63	**130.9**	**86.4**	1.0	0.66	0.01	n.a.	n.a.	n.a.	n.a.	Tested neg. for *ABCD1* mut
120	HSP	**♀**	37	101.7	**91.3**	1.03	0.90	0.01	n.a.	n.a.	n.a.	n.a.	Tested neg. for *ABCD1* mut
121	HSP	**♀**	55	108.4	**117.0**	1.28	1.08	0.012	**351**	92	61	158	Tested neg. for *ABCD1* mut
122	HSP	**♂**	50	82.8	**96.1**	1.43	**1.16**	0.017	217	**136**	58	107	Tested neg. for *ABCD1* mut
124	HSP	**♀**	47	110.4	**87.3**	**2.65**	0.79	0.024	**514**	**1501**	31	102	Tested neg. for *ABCD1* mut
129	frontal PSP	**♂**	61	50.0	70.7	1.19	**1.42**	0.024	198	**139**	42	154	Tested neg. for *ABCD1* mut
174	CADASIL	**♀**	53	105.9	**90.1**	0.95	0.85	0.009	**229**	60	86	121	Tested neg. for *ABCD1* mut
176	SCA3	**♂**	53	101.1	**87.8**	1.02	0.87	0.01	**244**	**236**	48	159	Tested neg. for *ABCD1* mut

CADASIL: Cerebral Autosomal Dominant Arteriopathy with Subcortical Infarcts and Leukoencephalopathy = caused by autosomal dominant inherited mutations in the *NOTCH3* gene; HSP: hereditary spastic paraplegia; mut.: mutations; n.a.: not analyzed; PSP: progressive supranuclear palsy an atypical form of Parkinson's diseases; SCA3: spinocerebellar ataxia type 3 or Machado-Joseph-Disease caused by autosomal dominant inherited CAG repeats in the *ATXN* gene; SPG4: spastic paraplegia type 4 = caused by autosomal dominant inherited mutations in the *SPAST* gene, SPG31: spastic paraplegia type 31 = caused by autosomal dominant inherited mutations in the *REEP1* gene; SPG39: spastic paraplegia type 39 = caused by autosomal recessive inherited mutations in the *PNPLA6* gene; TG: triglycerides; WMD: white matter disease or leukoenzephalopathy.

The bold marked values were above our preset cutoff values C26:0/C22:0 > 0.04; C24:0/C22:0 > 1.11; C22:0 > 115 µmol/l; C24:0 > 85 µmol/l; C26:0 > 1.9 µmol/l (compare Fig. 1). Lipid values elevated above the reference values are marked in bold.

### Statistics

Statistics were performed using SPSS 25 (IBM, Armonk, NY), including receiver operating curve (ROC) analysis. The Shapiro–Wilk Test tested Gaussian distribution due to 3 < n < 3000. All Gaussian variables were tested using the two-sided t-test, the Mann–Whitney-U-test tested non-Gaussian variables, and the chi-square test analyzed gender using a two-sided exact p-value. Bonferroni correction was applied to correct for multiple testing with the corresponding alpha-level specified below the Tables. Bivariate correlations were analyzed using Karl Pearson's coefficient for Gaussian distributed variables only and Spearman's rank correlation coefficient for non-Gaussian distributed variables. For the correlation, including the mutation types (a dichotomous variable), a point-biserial correlation was used. For all correlations, a 2-tailed significance level of *p* < 0.002 was considered to be statistically significant calculated using Bonferroni correction, with a total of 25 correlations analyzed^12^.

## Results

### VCLFA analysis

In a first step, a total of n = 1,953 VLCFA samples were tested for elevated VLCFA in the Institute for Clinical Chemistry and Pathobiochemistry of the University Hospital Tübingen since 01/2008 (Fig. 1). This analysis led to a total of 45 positive cases with at least one elevated VLCFA results. All 45 cases were genetically tested. In 30 cases, an *ABCD1* gene mutation was identified and these cases were included prospectively into the patient cohort. In 15 cases, no *ABCD1* gene mutation or another underlying disease was identified. To evaluate possible lipid-associated factors, these cases are listed together with their individual VLCFA and corresponding blood lipid levels in Table 2. All *ABCD1* gene mutations found are listed in Supplementary Table 1.

We observed elevated VLCFA in obviously lipemic sera and, therefore, consecutively analyzed the lipid profiles in VLCFA samples. We found that in all cases (n = 10) with elevated VLCFA levels, cholesterol and/or triglyceride levels, if available, were elevated. However, when using the ratios (relating to C22:0), not one single C26:0/C22:0 ratio and only three C24:0/C22:0 ratios remained elevated. Remarkably, two of them had very low C22:0 values.

**Figure 3:**
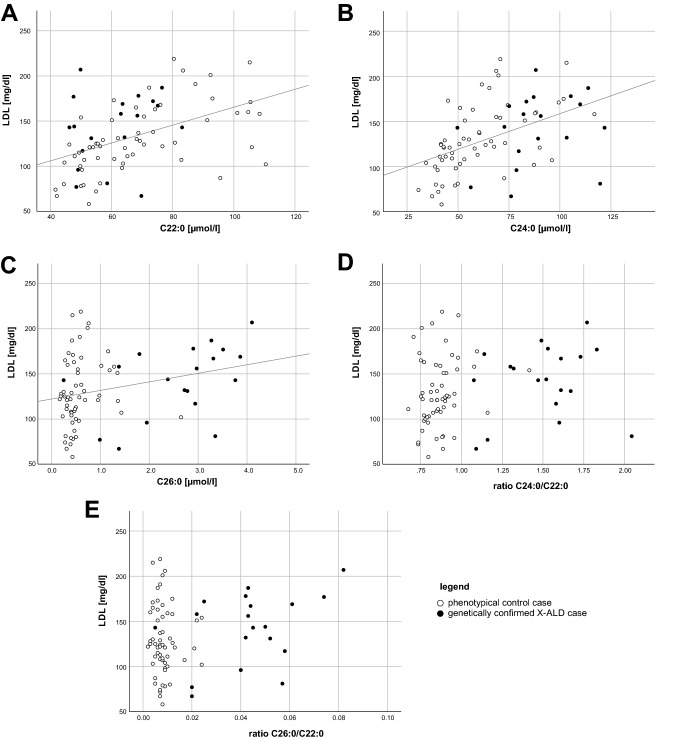
Correlation of LDL levels and VLCFA values. LDL levels in mg/dl are shown in scatter blots with the VLCFA values C22:0 (**A**), C24:0 (**B**), C26:0 (**C**) and the ratios C24:0/C22:0 (**D**) and C26:0/C22:0 (**E**). The phenotypical control cases are shown by white circles, the X-ALD cases by filled black circles with each circle representing a single patient. Spearman’s rank correlation coefficient indicated a significant correlation for LDL with C22:0 (r_s_ = 0.521; n = 89; *p* < 0.001), C24:0 (r_s_ = 0.548; n = 89; *p* < 0.001), and C26:0 (r_s_ = 0.415; n = 89; *p* < 0.001), but not for the ratios C24:0/C22:0 (r_s_ = 0.305; n = 89; *p* = 0.004), and C26:0/C22:0 (r_s_ = 0.262; n = 89; *p* = 0.013). A 2-tailed significance level of *p* < 0.002 was considered to be statistically significant (Bonferroni correction—see “Methods” section for details). Further correlations can be found in Table 4 together with the related p-values and the corresponding scatter blots for total cholesterol and VLCFA can be found in the supplementary material.

VLCFA analyses identified significant differences on a group level in VLCFA levels (C22:0, C24:0, C26:0, and both ratios) when comparing the X-ALD cases with the phenotypic controls. When comparing the X-ALD with the increased VLCFA Non-X-ALD group (n = 15), significant differences were also found. Further details are shown in Table 3. Spearman’s coefficient indicated that there was a significant positive correlation between total serum cholesterol (driven by serum LDL levels) and VLCFA values. Detailed results presented in Table 4 show that C22:0, C24:0 and C26:0 levels are significantly correlated to LDL levels but the associations are lost when values were analyzed as a ratio to C22:0. Scatter blots differentiating between X-ALD and Non-X-ALD cases are shown in Fig. 3 for LDL and VLCFA. Further scatter blots for total cholesterol and VLCFA values including the values of consecutive visits can be found in the supplementary material (supplementary Fig. 2). There was no positive correlation using a point-biserial coefficient between VLCFA levels and mutation type (missense vs. frameshift/deletion/nonsense) C22:0 (r(32) = − 0.10, *p* = 0.592) and C24:0 (r(32) = − 0.06, *p* = 0.975), C26:0 (r(32) = − 0.06, *p* = 0.973) nor the ratios C24:0/C22:0 (r(33) = 0.11, *p* = 0.537) and C26:0/C22:0 (r(33) = 0.05, *p* = 0.766).

**Table 3:**
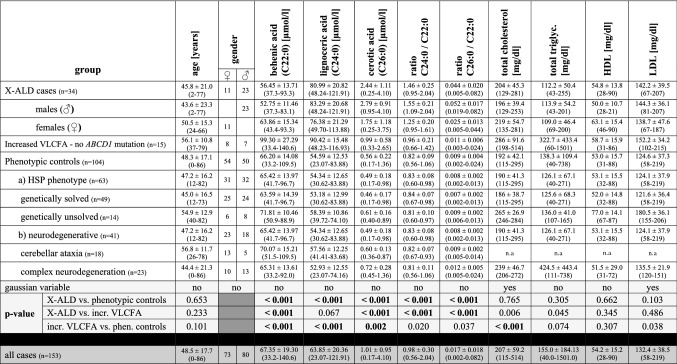
VLCFA and plasma cholesterol levels in X-ALD patients and Non-X-ALD cases (increased VLCFA cases and phenotypic controls).

Data are presented as mean ± standard deviation (range (maximum-minimum value)). The Shapiro–Wilk Test tested Gaussian distribution due to 3 < n < 3,000. All Gaussian variables were tested using the two-sided t-test and the Mann–Whitney-U-test tested non-Gaussian variables. Bonferroni correction for all tested parameters considered all p-values below an alpha of 0.5% (10 items) to be statistically significant, showing in bold the significant findings. All mutations of the X-ALD patients can be found in supplementary Table 1.

incr.: increased; n.a.: not analyzed; phen.: phenotypic

**Table 4:** Correlations of VLCFA and blood lipid levels.

	C22:0	C24:0	C26:0	Ratio C24:0/C22:0	Ratio C26:0/C22:0
total cholesterol	***p < 0.001*** r_s_ = 0.638; n = 92	***p < 0.001*** r_s_ = 0.521; n = 92	***p < 0.001*** r_s_ = 0.365; n = 92	*p* = 0.023 r_s_ = 0.237; n = 92	*p* = 0.067 r_s_ = 0.192; n = 92
LDL	***p < 0.001*** r_s_ = 0.521; n = 89	***p < 0.001*** r_s_ = 0.548; n = 89	***p < 0.001*** r_s_ = 0.415; n = 89	*p* = 0.004 r_s_ = 0.305; n = 89	*p* = 0.013 r_s_ = 0.262; n = 89
HDL	*p* = 0.085 r_s_ = 0.183; n = 89	*p* = 0.470 r_s_ = 0.077; n = 89	*p* = 0.712 r_s_ = -0.040; n = 89	*p* = 0.682 r_s_ = 0.044; n = 89	*p* = 0.342 r_s_ = -0.102; n = 89
TG	*p* = **0.001** r_s_ = 0.341; n = 90	*p* = 0.060 r_s_ = 0.199; n = 90	*p* = 0.359 r_s_ = 0.098; n = 90	*p* = 0.744 r_s_ = 0.035; n = 90	*p* = 0.800 r_s_ = 0.027; n = 90

Bivariate correlations were analyzed using Spearman's correlation coefficient due to the non-Gaussian distributed very long chain fatty acids values, TG, and HDL. A 2-tailed significance level of *p* < 0.002 was considered to be statistically significant (highlighted in bold), which was calculated using Bonferroni correction (total of 25 correlations analyzed—including mutation types as described in the “Results” section).

HDL: high-density lipoproteins; LDL: low-density lipoproteins; TG: triglycerides.

### Receiver operating curve analysis

In a second step (definition of the cohort in the “Methods” section—redefining diagnostic cutoffs), a total of 153 cases were analyzed to evaluate VLCFA values for sensitivity and specificity by ROC analysis (see Fig. 2). For eleven participants, consecutive VLCFA measurements (up to eight visits per patient) were available. Those were analyzed separately; see Supplementary Material for results.

### Analysis of current cutoffs

Our previous in-house cutoff values (compare Fig. 1) were evaluated for their sensitivity and specificity in genetically confirmed X-ALD cases exclusively. For C24:0 (< 85 µmol/l) the sensitivity was 36.4% with 88.2% specificity and a PPV of 48%, and for C26:0 (< 1.9 µmol/l)^13^ the sensitivity was 66.7% with 99.2% specificity and a PPV of 92%. For the ratio C24:0/C22:0 (< 1.11)^13^ the sensitivity was 90.9% with 96.6% specificity and a PPV of 91% for C26:0/C22:0 (< 0.04) the sensitivity was 66.7% with 100% specificity and a PPV of 96%.

### Analysis of proposed cutoffs

To increase sensitivity, we evaluated new cutoff values in a cohort as defined in the “Methods” section using ROC analysis (shown in Fig. 2B). This analysis resulted in the following new cutoff values: C22:0 < 105 µmol/l, C24:0 < 92 µmol/l, C26:0 < 1.2 µmol/l, ratio C24:0/C22:0 (< 1.0) and ratio C26:0/C22:0 (< 0.02). We found substantially increased sensitivity values particularly for C26:0 (+ 15.1%) and the ratios C24:0/C22:0 (+ 6.1%) and C26:0/C22:0 (+ 24.2%) (see Table 5) using the new cutoff values but also an increase by n = 3 false-positive findings (total n = 18) when taking all VLCFA values into account. On an individual level, the following VLCFA values were elevated in Non-X-ALD patients resulting in false-positive findings with the possibility of more than one increased VLCFA value per patient: C22:0 n = 9, C24:0 n = 7, C26:0 n = 5, and the ratios C24:0/C22:0 n = 6 and C26:0/C22:0 n = 6.

**Table 5:** Proposed optimized cutoff values.

	Cutoff	Sensitivity (%)	Specificity (%)	PPV (%)
C22:0	< 105 µmol/l	0.0	92.4	0.0
C24:0	< 92 µmol/l	24.2	94.1	50.0
C26:0	< 1.2 µmol/l	81.8	94.1	82.1
ratio C24:0/C22:0	< 1.0	97.0	94.1	83.8
ratio C26:0/C22:0	< 0.02	90.9	95.0	80.6

All values equal or above the mentioned values are considered to be abnormal.

PPV: positive predictive value.

## Discussion

This study is the first using only genetically confirmed X-ALD cases to redefine cutoff values. Previously published studies (compare Table 1) used clinical X-ALD diagnosis or heterogeneous groups with partially genetically confirmed cases for retrieval of cutoff values. These adjusted cutoffs will lead to increased sensitivity for the detection of X-ALD in neurodegenerative patients if routine serum lipid levels are within normal ranges, as these are important in identifying false-positive elevated VLCFA levels.

The analysis of VLCFA is only requested in patients when the clinician considers X-ALD as a potential differential diagnosis for the underlying disease. In contrast to previous studies^10^, this study did, therefore, not include a large number of healthy controls but focused on phenotypical controls mimicking the X-ALD phenotype. Additionally, only genetically tested positive *ABCD1* mutation carriers were considered to be X-ALD cases. By analyzing receiver operating characteristic (ROC) of VLCFA levels in genetically confirmed cases, we show that the serum concentration of C26:0 was superior to C24:0 (Table 5 and Fig. 2) for the detection of X-ALD well in line with previous reports^14^. However, considering the ratio of VLCFA in our cohort we found that the ratio of C24:0/C22:0 showed lower specificity than the ratio of C26:0/C22:0 but C24:0/C22:0 showed a higher sensitivity for detection of genetically confirmed *ABCD1*-positive patients, compared to the ratio C26:0/C22:0 (Table 5 and Supplementary Table 3). There was an increase in false-positive results by 16.7% with the new cutoff values.

VLCFA are elevated in all male X-ALD patients regardless of age, disease duration, metabolic status, or clinical symptoms^15^. Vegetarian diet in healthy individuals seems to influence VLCFA levels shifting values towards those found in X-ALD patients^16^. We could show a significant positive correlation of VLCFA levels with a total cholesterol level driven by the LDL level in all patients and controls. There was no correlation between VLCFA levels and the ratios (C24:0/C22:0 and C26:0/C22:0). VLCFA tests are only performed in neurological patients if X-ALD is considered a relevant differential diagnosis (e.g. in our specialty clinic for hereditary spastic paraplegias^17^, where VLCFA are routinely tested). Therefore, it is not surprising to not have a single neurologically healthy patient with elevated VLCFA levels in all 1,953 samples tested since 2008. Some *ABCD1*-negative neurodegenerative cases with mostly spastic para-/or tetraplegia displayed an elevation of single VLCFA.

Two genetically confirmed *ABCD1* mutation carriers were included in the cohort for ROC analysis despite negative VLCFA results using the current cutoffs. Two of the three cases which were not recognized by a cutoff ≥ 0.02 for the ratio of C26:0/C22:0 presented with the mutation c.1816 T > C, p.S606P (both females), shown to be pathogenic in at least seven patients according to the ALD mutation database (http://adrenoleukodystrophy.info^18–21^. A skewed level of X-inactivation in females is thought to cause this effect. The mutation type and VLCFA levels did not show a correlation in our analysis. While the measurement of total C26:0 is highly specific in male patients (100%), measurement of VLCFA can only detect about 85% of heterozygous female carriers^22^. Therefore, this effect could be gender-dependent rather than mutation-specific or could depend on X-chromosomal inactivation.

A previous report describes the finding of 1-hexacosanosyl-2-lyso-sn-3-glycero-phophatidylcholine (26:0-lyso-PC) in dried blood spots as a new biomarker for X-ALD screening in newborns (Hubbard et al. 2009). Validating this biomarker in 1,000 samples from newborns, Hubbard et al. found that the method is highly sensitive and accurate in identifying individuals with X-ALD (Hubbard et al. 2009). Applying the new method to plasma samples from adult patients, Huffnagel et al. show that 26:0-lyso-PC in dried blood spots was elevated in all women with X-ALD suggesting that this biomarker may identify women with X-ALD previously not found by analyzing plasma VLCFAs (Huffnagel et al. 2017).

Our observations that both total cholesterol and LDL-cholesterol levels, but not triglycerides levels, show a significant correlation to VLCFA C24:0 and C26:0 levels (Table 4) indicate that low-density lipoprotein contains lipids carrying VLCFA. LDL is composed of approximately 50% cholesterol, most of it is esterified with fatty acids and 20% phospholipids containing fatty acids. The questions of whether cholesterol-esters of X-ALD patients show increased content of VLCFA remains. Several publications report a highly increased content of VLCFA in cholesterol-esters obtained from the brain of X-ALD patients^23,24^. Their data show that VLCFA incorporation into cholesterol-esters is reduced, but more importantly, the reverse reaction (i.e. hydrolysis) is prolonged, indicating that VLCFA are trapped.

Similarly, phospholipids from the white matter of X-ALD patients show elevated VLCFA content^25^. However, one study reports that the VLCFA fraction is not elevated in serum cholesterol-esters in X-ALD patients^14^. Our data indicate that LDL levels strongly correlate to all VLCFA. However, normalizing to C22:0 this elevation is no longer significant (*p* ~ 0.06). This is not surprising as if all VLCFA are elevated to a similar extent, their ratios will be normal. Thus, elevated LDL levels influence VLCFA levels but only barely C24:0/C22:0 and C26:0/C22:0 ratios. To detect hypercholesterolemia, we recommend the determination of the lipid status of X-ALD patients together with VLCFA levels in fasted samples. Since spontaneous samples (including fasted and unfasted samples) were used, we would also like to address the necessity of additional serial testing in patients with uncertain results.

A combination of the cutoff values (two ratios and C26:0) with a sum score did not improve the sensitivity or the specificity compared to the ratio C24:0/C22:0 alone. Even by optimizing cutoff values, we did not obtain a sensitivity of 100% with a high specificity of also 100%, being aware that reference ranges and cutoff values are linked to each other. The sensitivity increased with the addition of 37 samples from consecutive tests (see Supplementary Material): C24:0 by 14.4% to 36.6%; C26:0 by 3.9% to 85.7%, while the sensitivity for the ratios C24:0/C22:0 and C26:0/C22:0 remained virtually unchanged. The new cutoff (> 0.02 ratio C26:0/C22:0) missed one female patient with five follow-up measurements available: four were below 0.02 and one equal to 0.02. The C24:0/C22:0 ratio was above > 1.0 in all five measurements. Therefore, we suggest using the two VLCFA ratios (C24:0/C22:0 and C26:0/C22:0) with the adjusted cutoff values to differentiate X-ALD from Non-X-ALD patients. Despite our findings, any genetic testing covering the phenotype should include the *ABCD1* gene not to miss, especially female patients. In our cohort of 34 VLCFA measured X-ALD patients, one female carrying the previously discussed c.1816 T > C, p.S606P mutation would have been missed using the adjusted cutoff ratios. This is slightly better than the previously published rate of 15% false-negative test results in females^22^ due to the heterozygosity of *ABCD1* in females.

## Conclusion

The use of serum VLCFA are economical and established (despite being laboratory, method, and assay-specific) biomarkers suitable for the guidance of genetic testing matching the X-ALD phenotype. We suggest to use our new optimized cutoff values (Table 5), especially the two ratios (C24:0/C22:0 (< 1.0) and C26:0/C22:0 (< 0.02)), in combination with standard lipid profiles as a screening tool.

## Supplementary information

Supplementary Information 1.

## Data Availability

The data sets for this manuscript are not publicly available, because raw data regarding human subjects (e.g., genetic raw data, personal data) are not shared freely to protect the privacy of the human subjects involved in this study; no consent for open sharing has been obtained. Requests to access the data sets should be directed to Dr. Tim W. Rattay.

## Abbreviations

AMN: Adrenomyeloneuropathy
CADASIL: Cerebral Autosomal Dominant Arteriopathy with Subcortical Infarcts and Leukoencephalopathy
HDL: High-density lipoprotein
HSP: Hereditary spastic paraplegia
LDL: Low-density lipoprotein
NGS: Next-generation sequencing
PPV: Positive predictive value
PSP: Progressive supranuclear palsy
VLCFA: Very long chain fatty acids
ROC: Receiver operating curve
SCA3: Spinocerebellar ataxia type 3 or Machado-Joseph-Disease
SPG4: Spastic paraplegia type 4
SPG31: Spastic paraplegia type 31
SPG39: Spastic paraplegia type 39
TG: Triglycerides
WMD: White matter diseases
X-ALD: X-linked Adrenoleukodystrophy
