# Supplementary material for: Defining diagnostic cutoffs in neurological patients for serum very long chain fatty acids (VLCFA) in genetically confirmed X-Adrenoleukodystrophy
Source: Sci Rep. 2020 Sep 15;10:15093. doi: 10.1038/s41598-020-71248-8 (PMC7494896; doi:10.1038/s41598-020-71248-8)
Supplement: Supplementary file 1 — Supplementary Information 1. [file 41598_2020_71248_MOESM1_ESM.docx]

**Defining diagnostic cutoffs in neurological patients for serum very long chain fatty acids (VLCFA) in genetically confirmed X-Adrenoleukodystrophy**

Tim W. Rattay, MD^1,2^; Maren Rautenberg, PhD^3^; Anne S. Söhn, PhD^3^; Holger Hengel, MD^1,2^; Andreas Traschütz, MD, PhD^1,2^; Benjamin Röben, MD^1,2^; Stefanie N. Hayer, MD, PhD^1,2^; Rebecca Schüle, MD^1,2^; Sarah Wiethoff, MD, PhD^1,2^; Lena Zeltner, MD^1,4;^; Tobias B. Haack, MD^3,4^; Alexander Cegan, PhD^5^; Ludger Schöls, MD^1,2,4^; Erwin Schleicher, PhD^6,7,8#^, Andreas Peter, MD^6,7,8^

# Corresponding author

**Author affiliations:**

1. Department of Neurology and Hertie-Institute for Clinical Brain Research, University of Tübingen, Tübingen, Germany
2. German Center of Neurodegenerative Diseases (DZNE), Tübingen, Germany
3. Institute of Medical Genetics and Applied Genomics, University of Tübingen, Tübingen, Germany
4. Center of rare diseases (ZSE), University of Tübingen, Tübingen, Germany
5. Department of Biological and Biochemical Sciences, Faculty of Chemical Technology, University of Pardubice, Pardubice, Czech Republic
6. Institute for Clinical Chemistry and Pathobiochemistry/Central Laboratory, University of Tübingen, Tübingen, Germany
7. German Center for Diabetes Research (DZD), Tübingen, Germany
8. Institute for Diabetes Research and Metabolic Diseases (IDM) of the Helmholtz Centre Munich at the University of Tübingen, Tübingen, Germany

**#Correspondence to:**

Prof. Dr. Erwin Schleicher

Institute for Clinical Chemistry and Pathobiochemistry / Central Laboratory, University of Tübingen, Hoppe-Seyler-Str. 3, 72076 Tübingen, Germany

Email: erwin.schleicher@uni-tuebingen.de

Tel.: +49 7071 29 80602

**Running title**: Defining diagnostic cutoffs for very long chain fatty acids

**SUPPLEMENTARY MATERIAL**

**Supplementary Table 1: All genetic mutations of the 34 X-ALD cases**

| **Patient ID** | **Mutation** |  | **Patient ID** | **Mutation** |
| --- | --- | --- | --- | --- |
| 1 | p.G51Afs*142 |  | 18 | c.1252C>T, p.R418W |
| 2 | c.1534G>A, p.G512S |  | 19 | c.887A>G, p.Tyr296C |
| 3 | c.889G>T, p.G279W |  | 20 | c.851C>T, p.S284L |
| 4 | c.1816T>C, p.S606P |  | 21 | c.421G>A, p.A141T |
| 5 | c.1816T>C, p.S606P |  | 22 | c.1772G>A, p. R519Q |
| 6 | c.234_242del, p.L79_81del |  | 23 | c.1252C>T, p.R418W |
| 7 | c.851C>T, p.S284L |  | 24 | c.1670T>G, p.V557G |
| 8 | c.1415delAG |  | 25 | c.1992G>A, p.W664* |
| 9 | c.1165C>T, p.R389C |  | 26 | c.1534G>A, p.G512S |
| 10 | c.593C>T, p.T198M |  | 27 | c.623_624del, p.V208Gfs* |
| 11 | c.2030C>A, p.G677D |  | 28 | c.119_147del, p.L49Rfs*145 |
| 12 | c.1849C>T, p.R617C |  | 29 | c.1661G>A, p.R554H |
| 13 | c.1667A>T, p.Q556L |  | 30 | c.1817C>T; p.S606L |
| 14 | c.411G>T, p.W137C |  | 31 | c.1233G>C, p.E411D |
| 15 | c.-40_483del (Ex1) |  | 32 | p.R153C |
| 16 | c.30G>A, p.W10* |  | 33 | c.421G>A, p.A141T |
| 17 | c.1165C>T, p.R389C |  | 34 | c.1992G>A; p.W664* |
| 20 | c.385dupG, p.Al129Gfs*66 |  |  |  |

For ID1 and ID32, the coding DNA sequence was not known due to an external laboratory DNA analysis and could not be specified.

**Supplementary Table 2: All VLCFA and plasma cholesterol levels including consecutive visits**

| **group** | | **gender** | | **behenic acid (C22:0) [µmol/l]** | **lignoceric acid (C24:0) [µmol/l]** | **cerotic acid (C26:0) [µmol/l]** | **ratio  C24:0 / C22:0** | **ratio  C26:0 / C22:0** | **total cholesterol [mg/dl]** | **total triglyc. [mg/dl]** | **HDL [mg/dl]** | **LDL [mg/dl]** |
| --- | --- | --- | --- | --- | --- | --- | --- | --- | --- | --- | --- | --- |
|  |  | **♀** | **♂** |  |  |  |  |  |  |  |  |  |
| X-ALD cases (v=71) | | 21 | 50 | 59.07 ± 14.69 (36.0-96.3) | 86.13 ± 23.96 (45.67-138.50) | 2.72 ± 1.67 (0.25-12.31) | 1.47 ± 0.27 (0.95-2.04) | 0.046 ± 0.026 (0.005-0.185) | 208 ± 41.9 (94-281) | 126.9 ± 74.19 (43.0-468.0) | 53.1 ± 13.5 (28-90) | 144.6 ± 41.4 (40-213) |
| increased VLCFA cases without *ABCD1* mutation (v=18) | | 9 | 9 | 97.61 ± 25.48 (33.4-140.6) | 91.75 ± 14.46 (48.23-116.93) | 1.03 ± 0.53 (0.33-2.65) | 0.99 ± 0.21 (0.66-1.42) | 0.012 ± 0.006 (0.003-0.024) | 276 ± 83.0 (198-514) | 347.3 ± 412.30 (60.0-1501.0) | 56.5 ± 16.5 (31-86) | 152.2 ± 30.9 (102-215) |
| Phenotypic controls (n/v=107) | | 55 | 52 | 66.81 ± 14.54 (33.2-109.5) | 55.21 ± 13.01 (23.07-90.45) | 0.57 ± 0.23 (0.17-1.36) | 0.83 ± 0.08 (0.56-1.06) | 0.009 ± 0.004 (0.002-0.024) | 193 ± 41.9 (115-295) | 138.6 ± 107.41 (40.0-738.0) | 52.6 ± 15.6 (31-88) | 125.5 ± 37.1 (58-219) |
| a) HSP phenotype (n/v =66) | | 32 | 34 | 66.45 ± 14.76 (41.7-107.5) | 55.37 ± 13.42 (30.62-90.45) | 0.52 ± 0.21 (0.17-1.35) | 0.83 ± 0.08 (0.60-0.98) | 0.008 ± 0.003 (0.002-0.018) | 191 ± 41.1 (115-295) | 126.9 ± 66.17 (40.0-271.0) | 52.7 ± 15.4 (32-88) | 125.1 ± 37.7 (58-219) |
| genetically solved (n/v =51) | | 25 | 26 | 64.17 ± 14.41 (41.7-96.7) | 53.85 ± 13.18 (30.62-83.88) | 0.48 ± 0.22 (0.17-1.35) | 0.84 ± 0.07 (0.67-0.98) | 0.008 ± 0.003 (0.002-0.018) | 188 ± 38.8 (115-295) | 126.5 ± 67.29 (40.0-271.0) | 51.7 ± 14.7 (32-88) | 122.7 ± 36.2 (58-219) |
| genetically unsolved (n/v =15) | | 7 | 8 | 74.18 ± 13.65 (50.9-107.5) | 60.53 ± 13.34 (39.72-90.45) | 0.63 ± 0.16 (0.40-0.89) | 0.82 ± 0.10 (0.60-0.97) | 0.009 ± 0.002 (0.006-0.013) | 265 ± 26.9 (246-284) | 136.0 ± 41.01 (107.0-165.0) | 77.0 ± 14.1 (67-87) | 180.5 ± 36.1 (155-206) |
| b) neurodegenerative (n/v =41) | | 23 | 18 | 67.40 ± 14.35 (33.2-109.5) | 54.96 ± 12.48 (23.07-83.68) | 0.67 ± 0.23 (0.36-1.36) | 0.81 ± 0.09 (0.56-1.06) | 0.010 ± 0.005 (0.005-0.024) | 239 ± 46.7 (206-272) | 424.5 ± 443.36 (111.0-738.0) | 51.5 ± 29.0 (31-72) | 135.5 ± 21.9 (120-151) |
| cerebellar ataxia (n/v =18) | | 13 | 5 | 70.07 ± 15.21 (51.5-109.5) | 57.56 ± 12.25 (41.41-83.68) | 0.60 ± 0.13 (0.36-0.87) | 0.82 ± 0.07 (0.67-0.93) | 0.009 ± 0.002 (0.005-0.014) | n/a | n/a | n/a | n/a |
| complex neurodegenerative (n/v =23) | | 10 | 13 | 65.31 ± 13.61 (33.2-92.0) | 52.93 ± 12.55 (23.07-74.16) | 0.72 ± 0.28 (0.45-1.36) | 0.81 ± 0.11 (0.56-1.06) | 0.012 ± 0.005 (0.005-0.024) | 239 ± 46.7 (206-272) | 424.5 ± 443.36 (111.0-738.0) | 51.5 ± 29.0 (31-72) | 135.5 ± 21.9 (120-151) |
| gaussian variable | | no | | no | no | no | no | no | yes | no | no | yes |
| **p-value** | X-ALD vs. phenotypic cont. |  | | **< 0.001** | **< 0.001** | **< 0.001** | **< 0.001** | **< 0.001** | 0.040 | 0.864 | 0.604 | 0.027 |
|  | X-ALD vs. incr. VLCFA |  | | **< 0.001** | 0.242 | **< 0.001** | **< 0.001** | **< 0.001** | **0.001** | 0.015 | 0.317 | 0.504 |
|  | incr. VLCFA vs. phen. cont. |  | | **< 0.001** | **< 0.001** | **< 0.001** | **0.002** | 0.007 | **< 0.001** | 0.021 | 0.467 | 0.018 |
|  | |  |  |  |  |  |  |  |  |  |  |  |
| all cases (v=196) | | 85 | 111 | 66.88 ± 18.94 (33.2-140.6) | 69.68 ± 23.94 (23.07-138.50) | 1.38 ± 1.43 (0.17-12.31) | 1.07 ± 0.35 (0.56-2.04) | 0.023 ± 0.024 (0.002-0.185) | 209 ± 54.8 (94-514) | 158.7 ± 176.41 (40.0-1501.0) | 53.3 ± 14.8 (28-90) | 135.8 ± 39.3 (40-219) |

In contrast to table 3, v represents the number of visits tested per group, whereas n represents the number of participants per group**.** Data are presented as mean ± standard deviation (range (maximum – minimum value)). The Shapiro-Wilk Test tested Gaussian distribution due to 3<n<3000. The two-sided t-test analyzed all Gaussian variables and the Mann-Whitney-U-test tested non-Gaussian variables. Bonferroni correction for all tested parameters considered all p-values below an alpha of 0.5% (10 items) to be statistically significant, showing in bold the significant findings.

Abbreviation: cont.: controls pheno.; phenotypic; v: visits

**Supplementary Table 3: Cutoff values tested with all X-ALD values (including the consecutive values)**

|  | **cutoff** | **sensitivity** | **specificity** |
| --- | --- | --- | --- |
| **C220:0** | <105 µmol/l | 0.0% | 92.0% |
| **C24:0** | <92 µmol/l | 38.6% | 92.0% |
| **C26:0** | <1.2 µmol/l | 85.7% | 92.3% |
| **ratio C24:0/C22:0** | <1.0 | 97.1% | 93.6% |
| **ratio C26:0/C22:0** | <0.02 | 90.0% | 95.2% |

All values equal or above the mentioned values are considered to be abnormal.

**Supplementary Figure 1: ROC-curve analysis of VLCFA values (including the consecutive ones)**

**
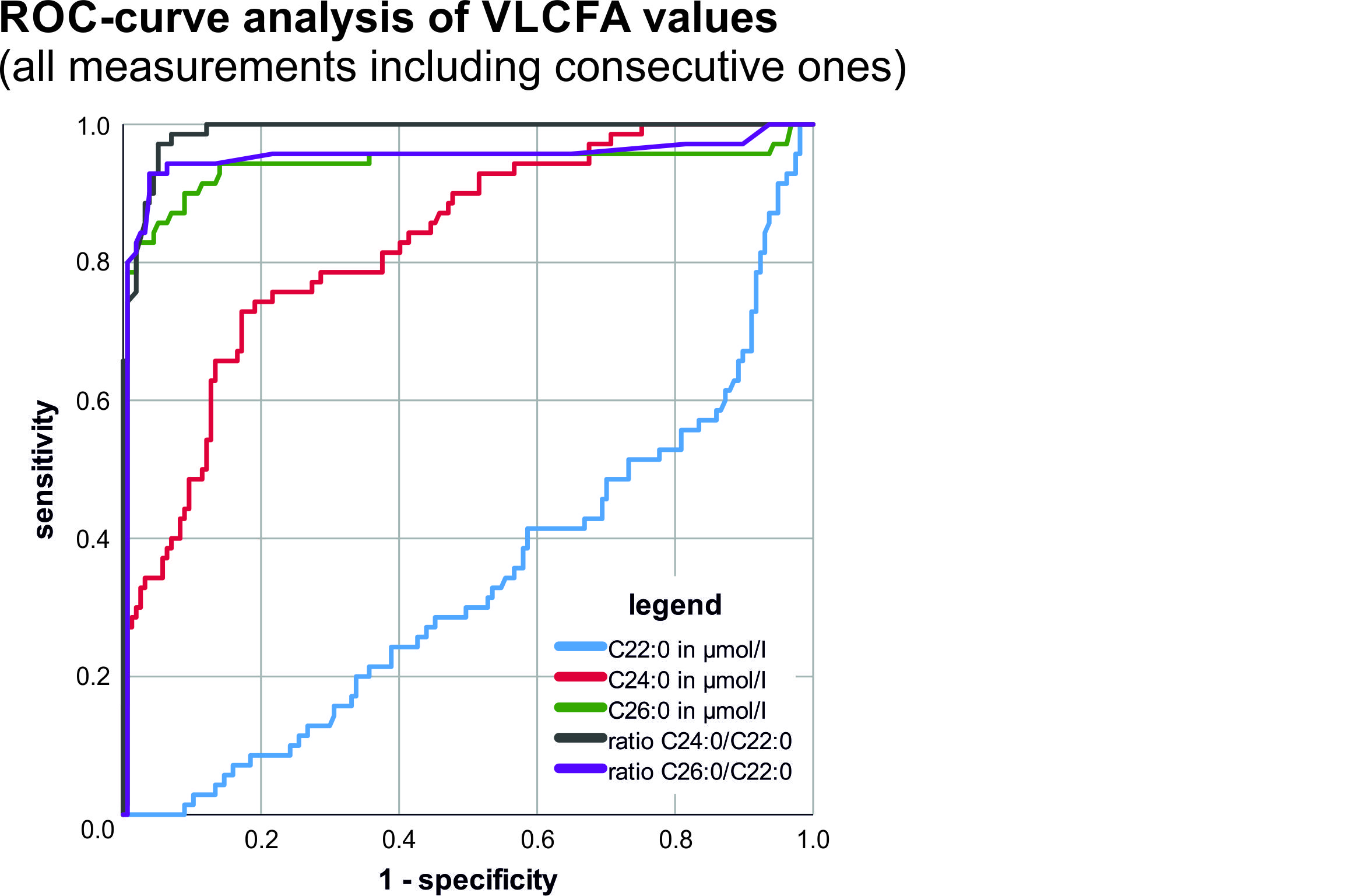
**

A ROC analysis of X-ALD (all 71 visits) vs. non-X-ALD cases (153 phenotypically similar cases) was performed using all five VLCFA measures (color coding see legend within the figure). Sensitivity and specificity were highest for the C24:0/C22:0 ratio followed by the C26:0/C22:0 ratio and the C26:0 absolute value.

**Supplementary Figure 2: Correlation of total cholesterol serum levels and VLCFA values**

**
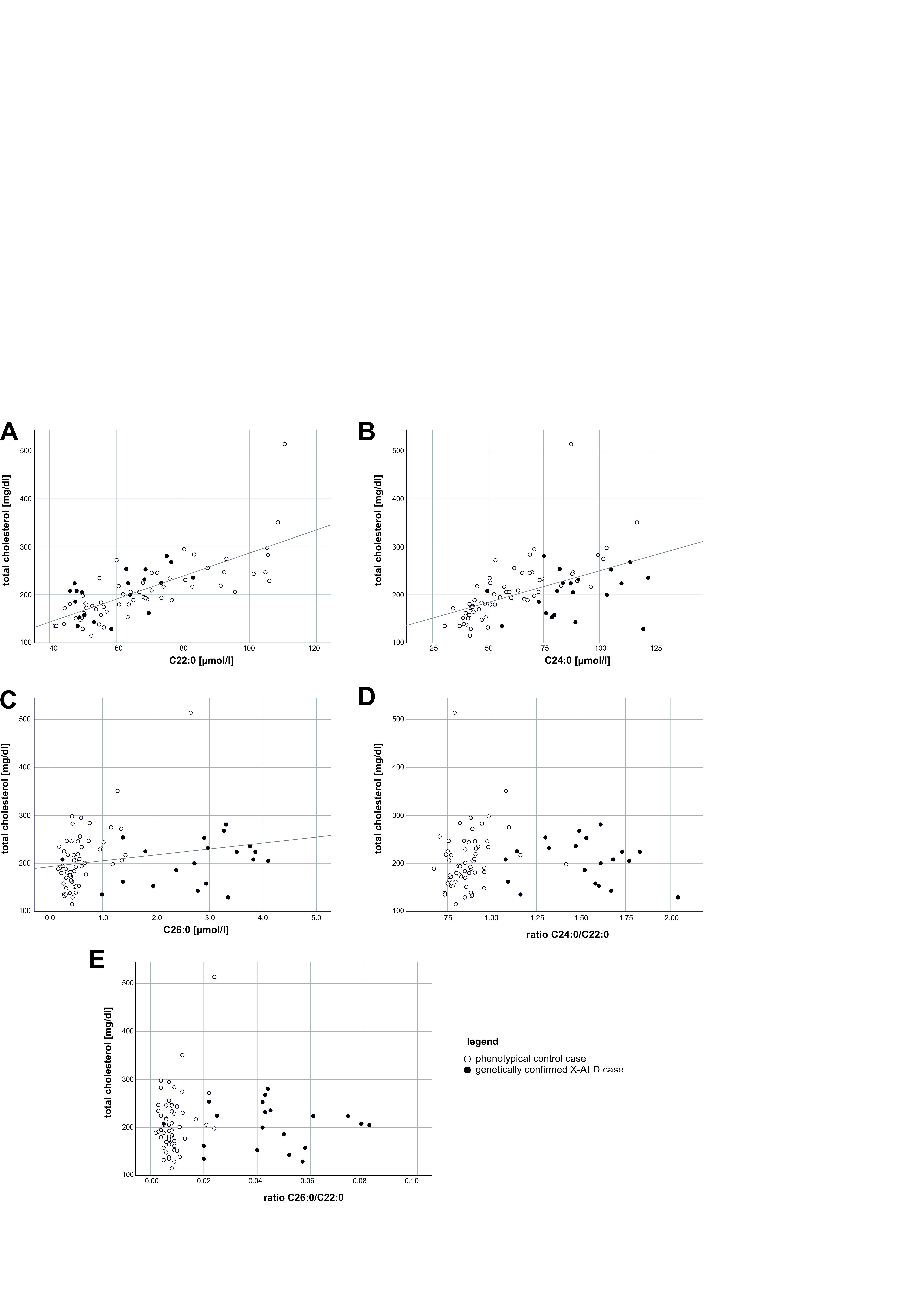
**

Total cholesterol levels in mg/dl are shown in scatter blots with the VLCFA values C22:0 (A), C24:0 (B), C26:0 (C) and the ratios C24:0/C22:0 (D) and C26:0/C22:0 (E). The phenotypical control cases are shown by white circles, the X-ALD cases by filled black circles with each circle representing a single patient. Spearman’s rank correlation coefficient indicated a significant correlation for total cholesterol with C22:0 (r_s_ = 0.638; n=92; p<0.001), C24:0 (r_s_ = 0.521; n=92; p<0.001), and C26:0 (r_s_ = 0.365; n=92; p<0.001), but not for the ratios C24:0/C22:0 (r_s_ = 0.237; n=92; p=0.023), and C26:0/C22:0 (r_s_ = 0.192; n=92; p=0.067). A 2-tailed significance level of p < 0.002 was considered to be statistically significant (Bonferroni correction – see methods section for details). Further correlations can be found in table 4 together with the related p-values.
